# Dysautonomia and Postural Orthostatic Syndrome of Hypocapnia in Long COVID Syndrome

**DOI:** 10.3390/jcm15186970

**Published:** 2026-09-09

**Authors:** Shane O’Brien, Thomas J. Butler, Edana Maher, Jack Allen, Tse Hue Chang, Christine O’Connor, Khaled Musameh, Conor Hayes, Laura Piggott, Ciara Scallan, Zara Cunningham, Carol Buckley, Deirdre B. Fitzgerald, Patrick D. Mitchell, Seamas C. Donnelly

**Affiliations:** 1 Tallaght University Hospital, D24 NR0A Dublin, Ireland; 2 School of Medicine, Trinity College Dublin, D02 PN40 Dublin, Ireland

**Keywords:** long COVID syndrome, dysautonomia, pulmonary rehabilitation, orthostatic intolerance

## Abstract

**Background**: Long COVID syndrome (LCS) affects at least 10% of COVID-19 survivors. Dysautonomia and orthostatic intolerance (OI) are recognised manifestations of long COVID syndrome (LCS). Postural orthostatic syndrome of hypocapnia (POSH) is among the most frequently identified physiological markers of orthostatic intolerance in this patient population. This study aims to evaluate the prevalence of OI and POSH in a cohort of patients with LCS and then treat patients with LCS and evidence of dysfunctional breathing and hypocapnia with targeted respiratory rehabilitation to evaluate the response in physiology and symptoms. **Methods**: Adults (>18 years) with a diagnosis of long COVID syndrome (LCS) underwent a NASA lean test (NLT) in our dedicated long COVID clinic. The NLT consists of measurements of respiratory rate, heart rate, blood pressure, oxygen saturation, end-tidal carbon dioxide (ETCO_2_), and symptoms taken over a 10 min period in the supine position, followed by repeated measurements in the leaning position. Patients with evidence of LCS, dysfunctional breathing and hypocapnia from the lean test were referred for targeted respiratory rehabilitation using the Papworth method. **Results**: A representative sample of patients with LCS (*N* = 50) underwent the NLT. Orthostatic symptoms were elicited in 50.0% of patients (*n* = 25) during the NASA Lean Test. Supine or orthostatic hypocapnia was identified in 64.0% of patients (*n* = 32), while postural orthostatic tachycardia and postural orthostatic hypotension were each observed in 32.0% of patients (*n* = 16). A total of 62.0% (*n* = 31) of patients with LCS who had persistent dyspnoea or evidence of OI as supported by supine or orthostatic hypocapnia were referred for targeted respiratory rehabilitation. Of the group referred, 41.9% (13 of the 31) completed respiratory rehabilitation. Comparing mean pre- and post-physiotherapy respiratory rehabilitation values, there was a significant improvement in the Malmö POTS score and the breathing pattern assessment tool (BPAT) (5.7 ± 1.9 vs. 2.1 ± 1.0, *p* < 0.0001) without a corresponding improvement in end-tidal CO_2_. **Conclusions**: These findings provide additional evidence supporting the presence of dysautonomia and OI in patients with long COVID syndrome (LCS). The NLT is a test for OI that can be easily performed in the clinic. Respiratory rehabilitation can improve symptoms related to dysfunctional breathing and OI in LCS. Although hypocapnia has been implicated in orthostatic cerebral vasoconstriction and was postulated as a treatable target in LCS, improvement in the symptoms that we observed were independent of changes in end-tidal CO_2_ on lean testing.

## 1. Background

At least 10% of COVID-19 survivors will develop long COVID syndrome (LCS) using conservative estimates [1]. Dysautonomia and orthostatic intolerance (OI) are recognised manifestations of long COVID syndrome (LCS) [2]. Postural orthostatic syndrome of hypocapnia (POSH) is among the most frequently identified physiological markers of orthostatic intolerance in this patient population [3]. Hypocapnia has been implicated in orthostatic cerebral vasoconstriction and has been demonstrated in patients with LCS. It has therefore been postulated as a potential treatable target in LCS [4].

LCS is defined internationally as the continuation or development of new symptoms 3 months after the initial SARS-CoV-2 infection, with these symptoms lasting for at least 2 months with no other explanation [5]. Proposed mechanisms include, but are not exclusive to, immune dysregulation, microbiota disruption, autoimmunity, clotting and endothelial abnormality, and dysfunctional neurological signalling [1]. Symptoms can be disabling, heterogeneous, and affect multiple organ systems. Dysautonomia and orthostatic intolerance (OI) have been reported in both myalgic encephalomyelitis/chronic fatigue syndrome (ME/CFS) and LCS [2]. OI is also a common finding in LCS. In a study by Monaghan et al., OI was identified in 66% of patients with LCS during active stand and 39% during tilt table test; however, POTS was identified in only 2% of that cohort [6]. One of the most frequent observations in patients with OI in ME/CFS is hypocapnia. Postural orthostatic syndrome of hypocapnia (POSH) has been used to describe orthostatic insufficiency with hypocapnia in patients with chronic fatigue syndrome as this was the most common marker of OI in a study by Natelson and colleagues [3]. Cerebral vasoconstriction has been demonstrated to occur during orthostatic intolerance (OI) in patients with ME/CFS. The application of CO_2_ rebreathing has been demonstrated to effectively reverse hypocapnia and alleviate orthostatic symptoms and has therefore been attributable to hyperventilation [4]. One hypothesis is that a centrally mediated cerebral vasoconstriction is responsible for the symptoms of OI, particularly in patients with symptoms of OI without evidence of cerebral hypoperfusion [7]. OI with hypocapnia is, however, independent of tachypnoea and hyperventilation in ME/CFS and may be secondary to hypernea or dysfunctional breathing given the normal respiratory rates found in patients with hypocapnia and OI [3]. Given the prevalence of OI and POSH in ME/CFS, the aim of our study is to evaluate the prevalence of OI and POSH in a cohort of patients with LCS. Our second objective is to identify individuals diagnosed with LCS exhibiting evidence of dysfunctional breathing and hypocapnia and treat those patients with targeted respiratory rehabilitation interventions to assess the response in symptoms and physiology. Therefore, our hypothesis is that dysfunctional breathing and hypocapnia in LCS is contributing to symptom burden and these may be targets amenable to treatment with respiratory retraining and pulmonary rehab.

## 2. Materials and Methods

### 2.1. Aim

This study aims to evaluate the prevalence of OI and POSH in our cohort of patients with LCS and treat patients with LCS and evidence of dysfunctional breathing and hypocapnia with targeted respiratory rehab to evaluate the response in physiology and symptoms.

### 2.2. Design

This was a retrospective single-centre observational study.

### 2.3. Methods

This study was performed in the long COVID outpatient clinic in Tallaght University Hospital, analysing routine data collected in the clinic. A representative sample of patients (*n* = 50) with a diagnosis of LCS aged >18 underwent a NASA lean test (NLT) in our clinic. The lean test was performed using a protocol developed by the National Aeronautics and Space Administration (NASA) [3,8]. With patients initially lying supine, baseline physiological measures—blood pressure, heart and respiratory rates, pulse oximetry and end-tidal CO_2_ [eTCO_2_] as assessed using a Microstream Capnograph (Capnostream 35 Portable Respiratory Monitor, Medtronics, Inc., Minneapolis, MN, USA)—were recorded twice, a minute apart. Patients were then asked to stand with their legs together approximately 6–8 inches from a wall and then to lean for 10 min while physiological measurements were taken every minute [3]. Patient symptoms were also recorded. Physiological definitions were as follows, as previously used by Natelson and colleagues [3]:

- Hypocapnia was defined as eTCO_2_ ≤ 32 mmHg in at least one measure: during supine rest and persisting through orthostatic challenge (supine hypocapnia) or only during orthostatic challenge (postural orthostatic syndrome of hypocapnia or POSH).
- Postural orthostatic tachycardia syndrome (POTS) was defined as a heart rate increase during orthostatic challenge of ≥30 beats per minute (BPM) compared with resting or ≥120 BPM.
- Orthostatic hypotension (OH) was defined as a drop of systolic pressure by ≥20 mmHg from baseline levels, occurring on at least 2 determinations after leaning.
- Tachypnoea was defined as a respiratory rate of 20 or more breaths per minute—either when supine or leaning [3].

Patients from this cohort with LCS, dysfunctional breathing and hypocapnia based on the lean test were referred for targeted pulmonary rehab. POTS was neither an inclusion nor an exclusion criterion. Patients were investigated to exclude alternative cardiac and respiratory diagnoses in the clinic, with laboratory blood tests, electrocardiograms, pulmonary function testing, radiology, and cardiac investigations as deemed appropriate by the treating team. Dysfunctional breathing is defined as breathing disorders where chronic changes in breathing patterns result in dyspnoea and other symptoms in the absence or excess of the magnitude of physiological respiratory or cardiac disease [9].

These patients were referred to a senior physiotherapist and reviewed with five face-to-face breathing retraining sessions.

Breathing retraining was administered using the Papworth method [10]. The Papworth method is a breathing and relaxation technique that aims to improve respiratory symptoms and quality of life in people with breathing pattern disorders. There are 5 different components to it:

- Breathing retraining aimed at achieving nasal and abdominal breathing, normal respiratory rate, tidal volume and inspiratory/expiratory ratio
- Education on respiratory pathology, normal breathing patterns and how external factors such as anxiety, stress and lifestyle influences can affect breathing pattern
- Relaxation training
- Integration of breathing and relaxation techniques into daily activities
- Home exercises

The following symptom scoring tools were administered pre- and post-rehabilitation to assess symptoms of OI, dysfunctional breathing, depression and anxiety, respectively: the Malmö POTS score [11], breathing pattern assessment tool (BPAT) [12], Nijmegen Questionnaire [13], Fatigue Severity Score [14] and hospital anxiety and depression scale (HADS) [15]. After the fifth session, the NLT and all questionnaires were completed again. A six-minute walk test was intended to be performed pre- and post-rehabilitation as an objective measure of functional exercise capacity; however, due to incomplete collection and missing data, complete walk-test distance data were not available for this cohort and were therefore not reported here.

The following parameters were recorded from our cohort:

- Demographics including age and sex;
- Past medical history;
- Date of index of COVID 19 infection and details regarding duration of illness and hospitalisation;
- Current medications;
- Laboratory results, pulmonary function testing results, and radiology results;
- Cardiac investigations including electrocardiogram, brain natriuretic peptide (BNP), echocardiogram, coronary angiogram and cardiac MRI in selected cases where the treating physician deemed this appropriate.

### 2.4. Data Analysis

All data were tested for normal distribution: Test for normal distribution = D’Agostino & Pearson test.

All data were normally distributed except for the HADS depression score.

To compare the baseline and final readings, a paired *t*-test was carried out, where a *p* < 0.05 was considered significant.

For normally distributed data (all but HADS depression scores), a parametric test was carried out (paired *t*-test). For HADS depression, a non-parametric Wilcoxon matched-pairs signed rank test was carried out.

All analyses were carried out using GraphPad Prism version 10.3.1.

## 3. Results

Patient characteristics and results for the overall cohort are summarised in Table 1.

Of the overall cohort (*n* = 50), the majority were female (76.0%, *n* = 38), with a mean age of 49.2 ± 12.8 years.

During baseline lean testing in the overall cohort (*N* = 50), orthostatic symptoms were elicited in 50.0% (*n* = 25) of patients on the NASA Lean Test. Supine or orthostatic hypocapnia was identified in 64.0% (*n* = 32) of patients, while postural orthostatic tachycardia and postural orthostatic hypotension were each observed in 32.0% of patients (*n* = 16).

A total of 62% (*n* = 31) of patients with LCS from the overall cohort of 50 who had persistent dyspnoea, had evidence of OI as supported by supine or orthostatic hypocapnia and were willing to engage in the physiotherapy programme were referred for targeted respiratory rehabilitation. A total of 41.9% (13 of the 31) of patients completed the programme and 51.6% (*n* = 16/31) of patients did not go on to have physio intervention due to symptom resolution (32.2% *n* = 10/31) or logistical issues (19.3% *n* = 6/31) that precluded them from attending for physio appointments. A total of 6.5% (*n* = 2/31) of patients started but did not complete the programme. There were six patients who reported dyspnoea but had no evidence of supine or orthostatic hypocapnia.

The demographics and characteristics of those who underwent respiratory physiotherapy intervention are summarised in Table 2. The mean age was 48.1 years. The majority were female 84.6% (*n* = 11) and all patients identified as Caucasian. The mean body mass index (BMI) was 31.7. Respiratory comorbidities were common: 53.8% (*n* = 7) had a diagnosis of asthma, and 15.4% (*n* = 2) had a diagnosis of chronic obstructive pulmonary disease (COPD).

**Figure 1:**
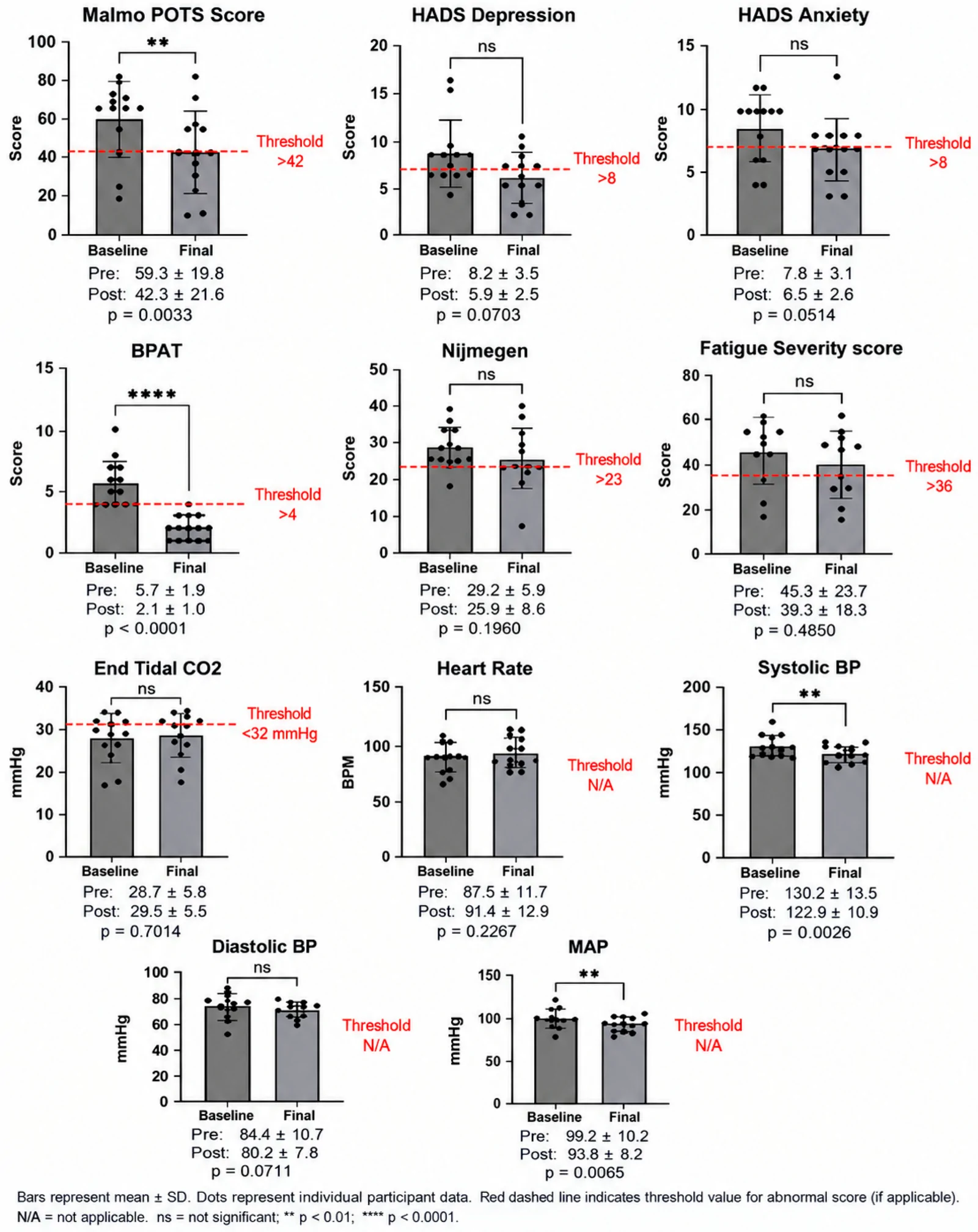
Summary of scores comparing mean values pre- and post-respiratory physiotherapy intervention with abnormal threshold values.

Comparing mean pre- and post-physiotherapy respiratory intervention values, there was a significant improvement in the Malmö POTS score (59.3 ± 19.8 vs. 42.3 ± 21.6, *p* = 0.0033), BPAT score (5.7 ± 1.9 vs. 2.1 ± 1.0, *p* < 0.0001), systolic blood pressure (Figure 1) and mean arterial pressure (99.2 ± 10.2 vs. 93.8 ± 8.2 mmHg, *p* = 0.0065). The mean post-treatment BPAT score fell below the threshold for abnormal breathing patterns. Changes were also observed in the HADS depression and anxiety scores, Nijmegen questionnaire scores, fatigue severity scores and diastolic blood pressure; however, these did not reach statistical significance. There was no significant change in end-tidal CO_2_ or heart rate following the intervention (Figure 1).

## 4. Conclusions

The NLT is an easy and quick test to perform in a clinic setting. Supine or orthostatic hypocapnia upon lean testing was the most prevalent indicator of orthostatic insufficiency in our cohort. The prevalence of orthostatic insufficiency symptoms is similar to what has been reported in larger multicentre cohorts. In one of the largest multicentre studies to date evaluating patients with long COVID using the NLT, the authors reported that 52% of patients developed symptoms during the NLT. In their study, 15% had an abnormal NLT; however, they did not evaluate patients with end-tidal CO_2_ measurements as we performed in our study [16].

One third of patients with LCS report dyspnoea [17]. Respiratory intervention in the form of a breathing retraining programme not only improved respiratory symptoms with an improvement in the BPAT score in our cohort but also significantly improved the Malmö POTS score, suggesting that respiratory retraining could have an impact on orthostatic insufficiency symptoms in patients with LCS.

Despite this, there was no difference between end-tidal CO_2_ values upon lean testing pre- and post-treatment. While supine or orthostatic hypocapnia upon lean testing may represent a marker of OI, it does not appear to change after respiratory rehabilitation.

One third of patients with POTS report dyspnoea, and the presence of hypocapnia can drive tachycardia in this cohort independent of hyperventilation [18,19].

Hypocapnia causes cerebral vasoconstriction and has been associated with reduced cerebral blood flow in patients with long COVID-POTS [20].

We had hypothesised that an improvement in hypocapnia may in turn improve cerebral blood flow in the cohort and therefore improve autonomic dysregulation; however, the symptom benefit appears to be independent of changes in the end-tidal CO_2_.

Limitations of this study include the relatively small sample size and the lack of a control group, which may limit the generalisability of our findings. Additionally, the observational nature of the study prevents definitive conclusions regarding causality. Of note is that patients were diagnosed with LCS, in some instances, by self-reported positive COVID-19 testing at home without verification, which is a standard practice in long COVID clinics; however, this is an important limitation as there may be cases within this cohort without confirmed acute COVID-19 and therefore LCS. The severity of COVID-19 illness and treatment regimens administered during acute COVID-19 illness were not recorded as part of this study. Acute disease severity may influence subsequent post-COVID manifestations, with greater long-term burden reported following more severe acute illness, particularly among those requiring hospitalisation or intensive care [21,22]. Similarly, treatments administered during the acute phase were not recorded, and there is evidence that acute therapeutic interventions, including antiviral treatment, may influence the subsequent risk of post-COVID condition [23]. Future prospective studies should therefore incorporate standardised, prospective recordings of acute disease severity and a predefined checklist of acute COVID-19 treatments, including treatment type, dose and duration, to allow their potential influence on subsequent post-COVID outcomes to be evaluated.

In total, 32.2% (*n* = 10 of 31) of the patients referred did not go on to have respiratory rehabilitation due to symptom resolution. Therefore, the cohort treated with respiratory intervention is reflective of those with persistent symptoms only.

In conclusion, our study further demonstrates that OI is common in patients with LCS and that respiratory physiotherapy is associated with significant improvements in symptoms of OI and respiratory symptoms of dysfunctional breathing in our cohort. Testing for OI with the NASA lean test is easy and quick to perform in a clinic setting. These findings highlight the therapeutic potential of targeted respiratory physiotherapy and rehabilitation in autonomic dysfunction and OI in LCS. Although hypocapnia has been proposed as a modifiable factor contributing to orthostatic cerebral hypoperfusion, symptom improvements following respiratory physiotherapy appear to occur independently of changes in end-tidal CO_2_ [4]. Improvements in orthostatic symptom burden occurred despite no significant change in end-tidal CO_2_, suggesting that the clinical benefits of respiratory rehabilitation may not be mediated solely through the correction of hypocapnia. Respiratory retraining may influence symptoms through other mechanisms, including improved breathing patterns, reduced ventilatory inefficiency and improved tolerance of orthostatic stress. The significant improvement in BPAT scores supports an improvement in breathing patterns despite the persistence of hypocapnia. These findings suggest that dysfunctional breathing and hypocapnia may represent related but partly independent physiological abnormalities in LCS.

## Figures and Tables

**Table 1:** Demographics and results for the entire cohort with LCS who underwent lean testing and measurement of end-tidal CO_2_.

Characteristic	Result
Total, *N*	50
Sex, female—*n* (%)	38 (76.0%)
Sex, male—*n* (%)	12 (24.0%)
Age, years—mean ± SD	49.2 ± 12.8
Orthostatic symptoms elicited during NLT—*n* (%)	25 (50%)
Dyspnoea	37 (74%)
Supine or orthostatic hypocapnia—*n* (%)	32 (64%)
Postural orthostatic tachycardia (POTS)—*n* (%)	16 (32%)
Orthostatic hypotension (OH)—*n* (%)	16 (32%)
Referred for targeted respiratory rehabilitation—*n*/*N* (%)	31/50 (62%)
Completed rehabilitation programme—*n*/*N* (%)	13/31 (41.9%)
Did not proceed: symptom resolution—*n*/*N* (%)	10/31 (32.2%)
Did not proceed: logistical issues—*n*/*N* (%)	6/31 (19.3%)
Started but did not complete programme—*n*/*N* (%)	2/31 (6.5%)

**Table 2:** Demographic and clinical characteristics of the patients at baseline treated with rehabilitation.

Characteristic	Results—Number (%)
Number of patients who completed rehabilitation	13
Age-years mean ± SD	48.1 ± 13.9
Female sex—no. (%)	Female 84.6% (*n* = 11)
Race—no. (%)	
- White	100%
- Asian	0%
- Black	0%
- Other	0%
Medical history	
- Asthma	7 patients (53.8%)
- COPD	2 patients (15.4%)
- Hypertension	1 patient (7.7%)
- Sinusitis	1 patient (7.7%)
BMI	31.7 ± 5.2
Smoking status	
- Current smoker	1 patient (7.7%)
- Never smoker	7 patients (53.8%)
- Ex smoker	5 patients (38.5%)

## Data Availability

The datasets generated and/or analysed during the current study are available from the corresponding author on reasonable request.
